# MicroRNA-377-3p released by mesenchymal stem cell exosomes ameliorates lipopolysaccharide-induced acute lung injury by targeting RPTOR to induce autophagy

**DOI:** 10.1038/s41419-020-02857-4

**Published:** 2020-08-19

**Authors:** Xuxia Wei, Xiaomeng Yi, Haijin Lv, Xin Sui, Pinglan Lu, Lijuan Li, Yuling An, Yang Yang, Huimin Yi, Guihua Chen

**Affiliations:** 1 Surgical and Transplant Intensive Care Unit, The Third Affiliated Hospital of Sun Yat-Sen University, No. 600, Tianhe Road, Tianhe District, Guangzhou, 510630 Guangdong, People’s Republic of China; 2 Department of Hepatic Surgery and Liver transplantation Center of the Third Affiliated Hospital, Organ Transplantation Institute, Sun Yat-sen University; Organ Transplantation Research Center of Guangdong Province, Guangdong province engineering laboratory for transplantation medicine, The Third Affiliated Hospital of Sun Yat-Sen University, No. 600, Tianhe Road, Tianhe District, Guangzhou, 510630 Guangdong, People’s Republic of China

**Keywords:** Mesenchymal stem cells, Respiratory tract diseases

## Abstract

Acute lung injury (ALI) and acute respiratory distress syndrome (ARDS) are the severe lung damage and respiratory failure without effective therapy. However, there was a lack of understanding of the mechanism by which exosomes regulate autophagy during ALI/ARDS. Here, we found lipopolysaccharide (LPS) significantly increased inflammatory factors, administration of exosomes released by human umbilical cord mesenchymal stem cells (hucMSCs) successfully improved lung morphometry. Further studies showed that miR-377-3p in the exosomes played a pivotal role in regulating autophagy, leading to protect LPS induced ALI. Compared to exosomes released by human fetal lung fibroblast cells (HFL-1), hucMSCs-exosomes overexpressing miR-377-3p more effectively suppressed the bronchoalveolar lavage (BALF) and inflammatory factors and induced autophagy, causing recoveration of ALI. Administration of miR-377-3p expressing hucMSCs-exosomes or its target regulatory-associated protein of mTOR (RPTOR) knockdown significantly reduced ALI. In summary, miR-377-3p released by hucMSCs-exosomes ameliorated Lipopolysaccharide-induced acute lung injury by targeting RPTOR to induce autophagy in vivo and in vitro.

## Introduction

Acute lung injury (ALI) is a severe inflammatory and damage diseases which can cause respiratory failure and compromised lung function, resulting in a mortality rate over 30%^1^. However, the effective strategy for treatment of ALI/ARDS has never been found yet. Thus, it is very urgent to find new therapeutic methods for treatment of ALI/ARDS.

Autophagy is a massive degradation pathway that plays key roles in various diseases^2^. However, whether autophagy exerts a protective or an active function in ALI remains unclear. A previous study showed that the enhancement of inflammation in mice with autophagy deficient macrophages^3^. Moreover, knockdown of two key autophagy initiation proteins (Beclin1 or Atg5) could reduce clearance and the accumulation of Pseudomonas in infected macrophages^4^. Additionally, inhibition of autophagy can augment lipopolysaccharide (LPS)-induced ALI through NF-κB signaling in human bronchial epithelial (HBE) cells^5^. Otherwise, recent reports indicate that autophagy plays a protective role during the occurrence of ALI. Meanwhile, Rapamycin (RAPA, autophagy activator) can induce the clearance of Pseudomonas from epithelial cells, while inactivation of autophagy can accumulates killing of Pseudomonas bacteria^6^. In addition, RAPA significantly alleviated lung inflammation and injury in ALI mice^7^. Moreover, LPS-induced lung injury and inflammatory responses can be diminished by RAPA^8^. Based on these backgrounds, autophagy display an active or detrimental role, depending on the pathogen and cell type affected during lung infection.

Mesenchymal stem cells (MSCs, cells of stromal origin) can be isolated from multiple human tissues which are an attractive cellular source for cell based therapy, including ALI^9^. Recently, various studies have indicated that administration of human umbilical cord-mesenchymal stem cells (hucMSCs) can alleviate the symptom of ALI; moreover, hucMSCs significantly improve survival, promote anti-inflammatory homeostasis and reduce oxidative stress in LPS-induced ALI in vivo^10,11^. Furthermore, low levels of TGF-β1 enhance fibronectin production in hucMSCs and extend survival time in a rat model of LPS-induced ALI^12^. However, the effect of hucMSCs on biological activity and clinical therapies remains largely unknown^13^.

Exosomes are small vesicles (30–200 nm in diameter) which released from various cell types and formed intracellularly in endocytic multivesicular compartments^14^. Exosomes contain proteins, mRNA and miRNA, and play a biological role in transmission of proteins, mRNA and miRNA. CD9, CD63 and CD81 are known to be characteristic markers of exosomes^15,16^. MSC-derived exosomes have been confirmed to play key roles in various rat models, including myocardial infarction^17^, acute lung injury^18^, acute liver failure^19^, acute kidney injury^20–22^ and nerve injury^23^. Although exosomes derived from MSCs have been successfully applied in improving pathology of ALI, the mechanism by which exosomes mediated the progression of ALI remains to be explored. In the current study, we found that exosomes released from hucMSCs induced autophagy in LPS-induced ALI. We showed that the activation of autophagy played a protective role in LPS-induced ALI. Specifically, overexpression of miR-377-3p, which contained in hucMSCs-exosomes, significantly suppressed lung pathology through down-regulation of RPTOR. Collectively, our study reveals that exosomes released from hucMSCs expressing miR-377-3p can alleviate LPS-induced ALI via activation of autophagy. Thus, our study provided a potentially effective therapeutic target for the treatment of ALI.

## Materials and methods

### ALI mice model

Male C57BL/6 mice (body weight 20 ± 2 g) aged 8 weeks were purchased from the Guangdong Medical Laboratory Animal Center, China (Certification number:SCXK 2018-0034)and raised in SPF with free access to food and water (50 ± 5% of humidity and 12-h day/night cycle). The mice were intratracheal instillation of 1.0 mg/kg of LPS (or with PBS as a control)^24^ after anesthesia by sodium pentobarbital (40 mg/kg). After 4 h, the mice were given hucMSCs or HFL-1 cells (2 × 10^5^) by intratracheal instillation. Mice were anaesthetized by sodium pentobarbital (40 mg/kg) after 72 h and the right lower lobe lung tissue of each group was collected for further study. All the animal experiments were approved by Ethics Committee of Sun Yat-sen University.

### Immunofluorescence assay

For lung tissues, the tissues were embedded in paraffin and cut into 4 µm sections. The slides were deparaffinized and rehydrated by xylene and different concentration of ethanol (100%, 95%, 80%, 70% and 50%). The antigen was repaired by using a microwave in 10 nm sodium citrate buffer (pH 6.0) for 10 min. After blocking with 1% goat serum and 0.1% Triton X-100 in PBS for 30 min at room temperature, LC3B (CST, 83506, 1:400) antibody was added to slide and incubated at 4 °C overnight. Slides were washed with PBS for three times and incubated with FITC conjugated secondary antibody at room temperature for 1 h and stained with DAPI for 5 min. Slides were examined under a microscope (Zeiss). For cells, the cells were fixed by 4% paraformaldehyde and blocked by 2% BSA for 30 min, the following steps were as same as the lung tissues stained by LC3B antibody.

### Cell culture

HucMSCs were provided by The Third Affiliated Hospital, Sun Yat-sen University and maintained in serum-free MesenCult-XF medium and FBS bases DMEM complete medium (Stemcell, Vancouver, Canada) at 37 °C and 5% CO_2_. Cells were passaged twice per week. HFL-1 cells were purchased from Bnbio and cultured in DMEM medium at 37 °C and 5% CO_2_. Cells were passaged three times per week. Human pulmonary alveolar epithelial cells (HPAEpiC) were purchased from Bnbio and cultured in F12 medium at 37 °C and 5% CO_2_. Cells were passaged twice per week.

### Flow cytometry

Total of 2 × 10^5^ hucMSCs were collected and suspended in 500 μl PBS. Monoclonal antibodies against CD19 (PE conjugated), CD73 (APC conjugated), CD90 (FITC conjugated), CD105 (PE-Cy7 conjugated), CD34 (PE conjugated) and HLA-DR (redFluor^TM^ 710 conjugated) were added to cells respectively and incubated for 45 min at 4 °C. After washing with PBS, cells were resuspended in 500 μl PBS and detected by FACS flow cytometer (BD Biosciences).

### Osteogenic and adipogenic differentiation

HucMSCs were cultured in osteogenic differentiation or adipogenic differentiation medium (Gibco) for 3 weeks. Cells of the third passages were seeded onto 12 well plates, after 12–16 h, the culture medium was replaced with differentiation medium every three days. Cells were washed with PBS and fixed with paraformaldehyde at 4% for 15 min and stained with oil red O or alizarin red S (Sigma-Aldrich) for 15 min. The images were taken by microscopy (Zeiss).

### Hematoxylin and eosin (H&E) staining

Lung tissues were fixed in 4% paraformaldehyde and embedded in paraffin and then cut into 4 µm sections. The slides were deparaffinized and stained with H&E. The inflammatory cell infiltration, bleeding, interstitial and alveolar edemas were observed under a light microscope and the lung injury was evaluated by a standard as follows:

0: No injury

1: slight injury

2: moderate injury

3: Serious injury

4: Very serious histological injury

### Collection of bronchoalveolar lavage fluid (BALF)

PBS (1 ml) was injected into lungs using a 2.5 ml syringe and the lavage was repeated three times. The lavage solution was centrifuged at 4 °C and 700 g for 5 min. The supernatant was collected and the total quantity of protein in BALF was measured using a BCA protein assay kit provided by Wuhan Boster Biology Technology.

### ELISA assay

The expression of MCP-1, IL-6, IL-1β and IL-17 in BALF of mice in each group was detected by ELISA kit according to instructions (Elabscience). The absorbance was measured using a microplate reader at 450 nm.

### Western blotting

Lung tissues or cells were lysed in RIPA buffer. The protein concentration was determined by BCA method. Total of 20 μg protein was loaded on 10%-12% SDS-PAGE. The protein was transferred to PVDF membrane and blocked in 5% BSA for 1 h at room temperature. After washing with 1 × TBST, the membrane was incubated with following antibodies, LC-3 (CST, 83506, 1:1000), p62 (CST, 88588, 1:1000), Beclin (CST, 3495, 1:1000) and GAPDH (abcam, ab8245, 1:10000) overnight at 4 °C. After washing with 1 × TBST for three times, the membrane was incubated with HRP conjugated secondary antibodies. The membranes were visualized with enhanced chemiluminescence.

### Cell transfection

Negative control shRNA, Rab27a-1 and Rab27a-2 shRNA plasmids were purchased from Genepharma (Shanghai, China). HucMSCs were transfected with control shRNA (5′GTCGAACGTCGTGAACCTACCATG3′), Rab27a-1 (5′CAUUAGACCUACGAAUAAA3′) or Rab27a-2 shRNA (5′GCUGCCAAUGGGACAAACA3′) by Lipofectermine 2000 according to manufacturer′s introduction. The miRNA mimics (5′AUCACACAAAGGCAACUUUUGU3′ and 5′AAAAGUUGCCUUUGUGUGAUUU3′) or inhibitor (5′ACAAAAGUUGCCUUUGUGUGAU3′) was transfected as above.

### Exosomes extraction and identification

Exosomes derived from hucMSCs and HFL-1 cells were isolated by the exosome isolation kit (Invitrogen, USA). The exosomes were identified under a transmission electron microscope (TEM) after negative staining with 3% (w/v) sodium phosphotungstate solution and ddH_2_O wash. The expression levels of exosome specific biomarkers, CD63 and CD9, were detected by Western blotting.

### Electron microscopy

Primary pulmonary epithelial cells (HPAEpiC) were fixed overnight at 4 °C in 2.5% glutaraldehyde with 1% tannic acid. The cells were washed 3 times in the sodium cacodylate buffer and dehydrated with graded steps of acetone (50%, 70%, 90% and 100%) and embedded in Spurr’ low viscosity media. Following polymerization at 70 °C for 12 h, 60 nm sections were cut on a Reighert-Jung Ultra cut Eultramicrotome (Leica Microsystems,) and picked up on copper grids. The grids were post-stained in uranyl acetate and bismuth subnitrate.

### MicroRNA microarray and data analysis

Exosome-miRNAs were prepared for array analysis (performed by Shanghai Biotechnology corporation, China). The miRNA expression was validated by real-time PCR for consistency.

### Real-time PCR

RNA from hucMSCs-exosomes or HFL-1-exosomes was extracted by Trizol reagent (Thermo). First stand cDNA was synthetized by Bestar^TM^ qPCR RT kit (DBI). The real-time PCR was performed by BestarTM qPCR Master Mix (DBI) in ABI real time PCR instrument (ABI). Primer sequences were listed in Table 1.

**Table1:** Primer sequences.

ID	Sequence (5′–3′)	Product length (bp)
GAPDH F	TGTTCGTCATGGGTGTGAAC	154
GAPDH R	ATGGCATGGACTGTGGTCAT	
U6 F	CTCGCTTCGGCAGCACA	96
U6 R	AACGCTTCACGAATTTGCGT	
All 1 R	CTCAACTGGTGTCGTGGA	
RPTOR.F	ACTGGAACCTACCTTTGGCTT	106
RPTOR.R	ACTGTCTTCATCCGATCCTTCA	
AKT1.F	TCCTCCTCAAGAATGATGGCA	181
AKT1.R	GTGCGTTCGATGACAGTGGT	
RHEB.F	TTGTGGACTCCTACGATCCAA	95
RHEB.R	GGCTGTGTCTACAAGTTGAAGAT	
hsa-miR-377-3p.RT	CTCAACTGGTGTCGTGGAGTCGGCAATTCAGTTGAGACAAAAGT	
hsa-miR-377-3p.F	ACACTCCAGCTGGGATCACACAAAGGCAACTT	
hsa-miR-130a-3p.RT	CTCAACTGGTGTCGTGGAGTCGGCAATTCAGTTGAGATGCCCTT	
hsa-miR-130a-3p.F	ACACTCCAGCTGGGCAGTGCAATGTTAAAAGG	
hsa-miR-188-5p.RT	CTCAACTGGTGTCGTGGAGTCGGCAATTCAGTTGAGCCCTCCAC	
hsa-miR-188-5p.F	ACACTCCAGCTGGGCATCCCTTGCATGGTGG	
hsa-miR-410-3p.RT	CTCAACTGGTGTCGTGGAGTCGGCAATTCAGTTGAGACAGGCCA	
hsa-miR-410-3p.F	ACACTCCAGCTGGGAATATAACACAGATGGC	

### Luciferase reporter assay

293 T cells at a density of 1 × 10^5^ per well were seeded onto 24-well plates, the pLUC-RPTOR-wild-type (WT) 3′ UTR or pLUC-RPTOR-mutant-type (MUT) 3′-UTR plasmid was co-transfected with the miR-377-3p mimic or miR-377-3p mimic negative control using Lipofectamine 2000 (Thermo Fisher Scientific, Inc.). After 36 h of transfection, the luciferase activities were measured at 560 nm by a luciferase reporter assay kit (Promega).

### Statistical analysis

Student presented as the means ± standard error of the mean and analyzed using SPSS software (version 20.0). In addition, 4 mice were performed in each group during the animal experiments. Statistical comparisons were made using a one-way ANOVA (for multi-group comparisons) or a two-tailed Student’s t test (between two groups). *P-*value <0.05 (*), *P-*value <0.01 (**) and *P*-value <0.001 (***) were considered to indicate statistically significant difference.

## Results

### HucMSCs protects LPS-induced ALI

To assess the effects of hucMSCs on ALI, we first identified characters and the differentiation potential of hucMSCs. As shown in Supplementary Fig. 1A, hucMSCs were CD73^+^, CD90^+^, CD105^+^, CD19^−^, CD34^−^ and HLA-DR^−^ by flow cytometry. Additionally, after culturing with osteogenic or adipogenic differentiation medium, hucMSCs were differentiated as stained by oil red O or alizarin red S (Supplementary Fig. 1B), indicating that hucMSCs we used to be well characterized and identified.

**Fig. 1:**
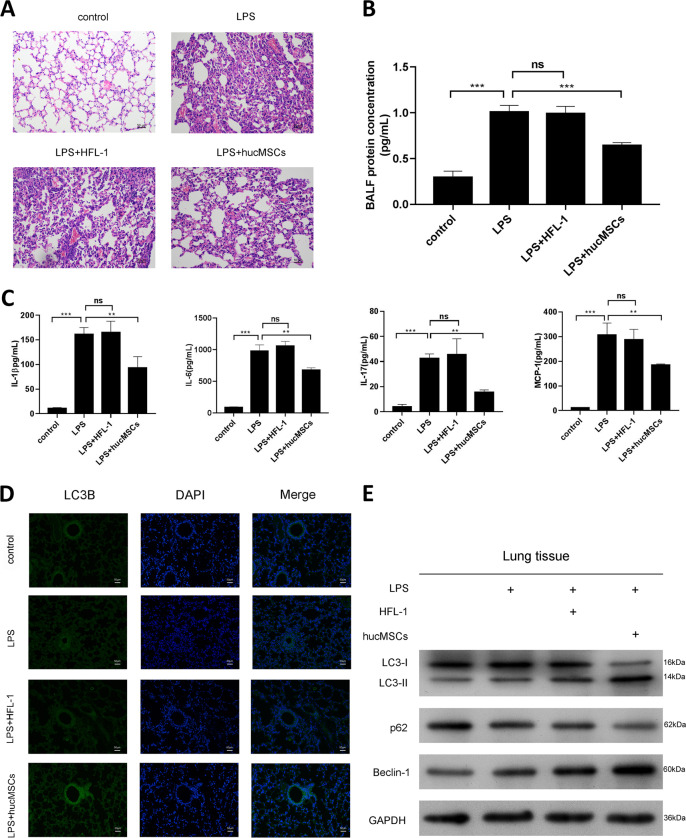
HucMSCs protects LPS induced acute lung injury (ALI). **a** C57BL/6 mice were intranasal instillated with LPS (1mg/kg weight). After 4h, total of 2×10^5^ hucMSCs or HFL-1 cells were subjected to mice through intratracheal instillation, 72h later, the lung tissues were collected and fixed for H&E staining. **b** BALF protein concentration was determined by BCA. *N* = 4. **c** The expression of IL-1β, IL-6, IL-17 and MCP-1 in BALF was tested by ELISA. *N* = 4. **d** The expression of LC3B was measured by IF. The respective images were shown. **e** The expression of LC3II/I, p62 and Beclin-1 was determined by Western blotting from lung tissues.

We established a mice ALI model by LPS and the pathological changes were shown by H&E staining. Histological evaluation of lung sections 4 h after the LPS instillation (solaibio L8880) revealed notable inflammatory cells infiltration (Fig. 1a). However, after intratracheal instillation of hucMSCs, the pathological changes in the lung tissues were relieved compared with HFL-1 cells.(Fig. 1a). We further detected the BALF protein concentration and found that LPS significantly increased BALF protein concentration, while hucMSCs reduced BALF protein concentration (Fig. 1b). The effect of hucMSCs on pulmonary inflammation was determined by ELISA and the results revealed that hucMSCs reversed LPS induced pulmonary inflammation (Fig. 1c). Moreover, the autophagy of lung tissues was detected by LC3 staining, (Fig. 1d), hucMSCs instillation enhanced autophagy. We also tested the autophagy markers, the expression of LC3 II/I and Beclin-1 were significantly up-regulated, while the expression of p62 was down-regulated by hucMSCs (Fig. 1e).

### Exosomes released by hucMSCs mediate hucMSCs function

To illustrate the molecular mechanism of hucMSCs protective effects on ALI, we detected whether exosomes released by hucMSCs may play essential roles in ALI prevention. As previous studies showed that the small GTPases Rab27a and Rab27b were critically required for exosomes secretion by Hela cells^25^ and the spontaneous secretion of exosomes from CD63-containing compartments was strongly decreased under Rab27a knockdown condition^26^. Here, we inhibited Rab27a by shRNA in hucMSCs (Supplementary Fig. 2A), and intratracheal instillation of Rab27a shRNA transfected hucMSCs to LPS treated mice after 4 h, the pathology of mice showed that knockdown of Rab27a abolished the protective roles of hucMSCs on LPS induced ALI (Supplementary Fig. 2B). Additionally, the BALF and inflammatory factors were also increased by shRab27a compared with shNC transfected hucMSCs cells under LPS treatment (Supplementary Fig. 2C, D). Moreover, the autophagy induced by hucMSCs was abolished by Rab27a knockdown (Supplementary Fig. 2E, F). Our data suggested that exosomes secreted by hucMSCs play protective roles in LPS induced ALI.

**Fig. 2:**
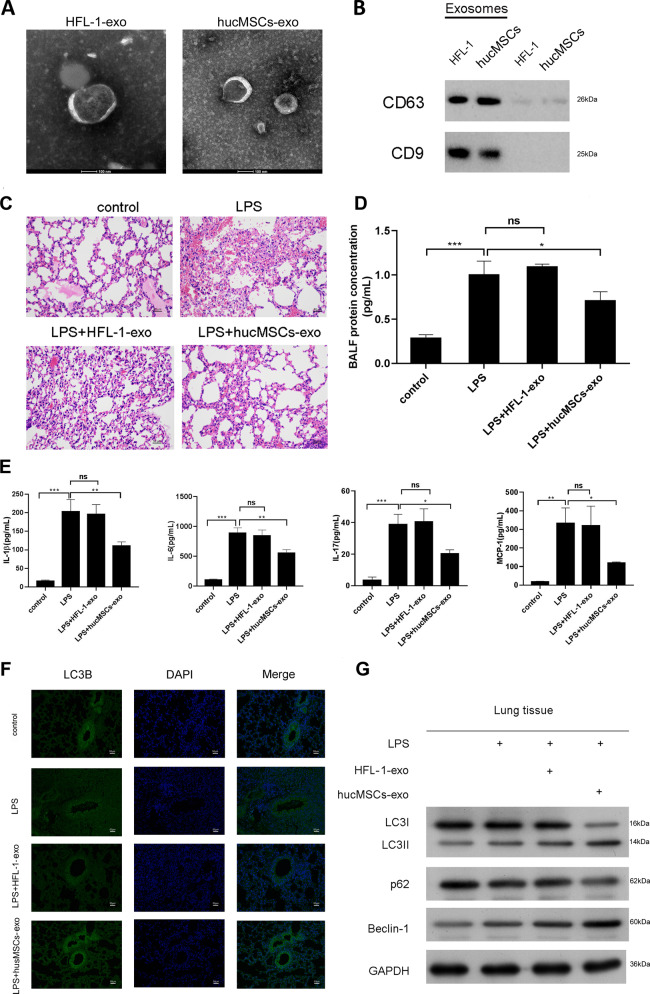
Exosomes mediates hucMSCs function. **a** Exosomes from hucMSCs or HFL-1 cells were extracted and identified by TEM. **b** The expression of exosomes markers, CD63 and CD9 were detected by Western blotting. **c** C57BL/6 mice were intranasal instillated with LPS (1mg/kg weight). After 4h, total of 50μg hucMSCs-exosomes or HFL-1-exosomes were subjected to mice through intratracheal instillation, 72h later, the lung tissues were collected and fixed for H&E staining. The result of pathology was presented. **d** BALF protein concentration was determined by BCA. *N* = 4. **e** The expression of IL-1β, IL-6, IL-17 and MCP-1 in BALF was tested by ELISA. *N* = 4. **f** The expression of LC3B was measured by IF. The respective images were shown. **g** The expression of LC3II/I, p62 and Beclin-1 were determined by Western blotting from lung tissues.

To illustrate the effects of exosomes secreted by hucMSCs on LPS induced ALI, we isolated exosomes from hucMSCs and HFL-1 cells respectively. In order to identify the characteristics of the exosomes, particle size and substantial shape of hucMSCs or HFL-1-exosomes were tested by TEM and Western blotting, the data showed both hucMSCs-exosomes and HFL-1-exosomes had a spheroid shape and positively expressed exosome markers CD63 and CD9 (Fig. 2a, b, Supplementary Fig. 4A, B).

Next, hucMSCs-exosomes or HFL-1-exosomes were intratracheal instillated to LPS treated mice after 4 h. The results showed that exosomes released by hucMSCs significantly alleviated LPS induced ALI in pathology (Fig. 2c, Supplementary Fig. 3), BALF concentration (Fig. 2d) and inflammatory factors (Fig. 2e). More importantly, exosomes secreted by hucMSCs significantly enhanced autophagy (Fig. 2f–g), while exosomes from HFL-1 cells did not show obvious effects on LPS induced ALI, suggesting that exosomes released by hucMSCs played important role in preventing LPS induced ALI.

### Exosomes ameliorates LPS induced inflammation in HPAEpiC by stimulating autophagy

**Fig. 3:**
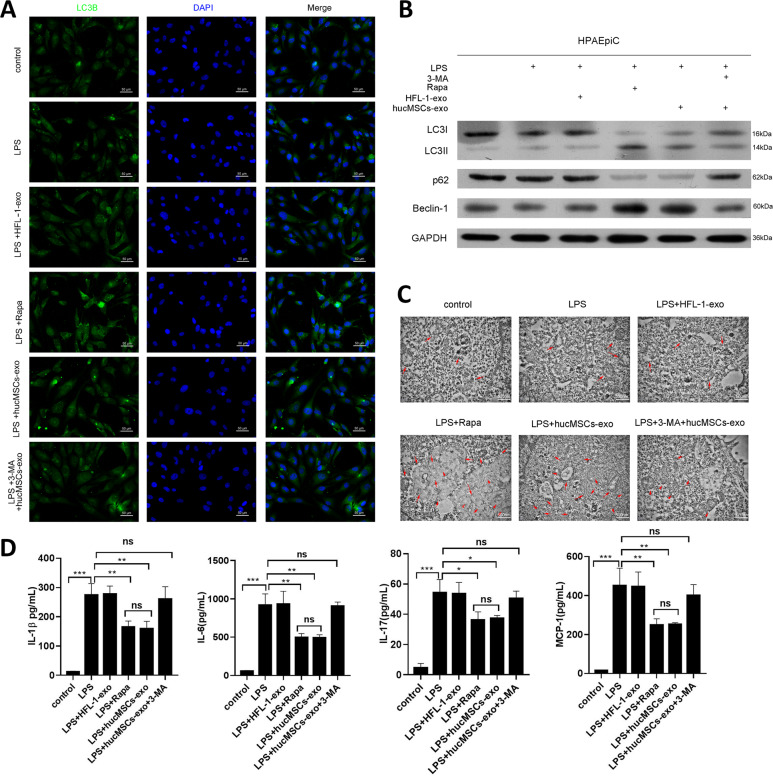
Exosomes alleviate LPS induced inflammation in primary pulmonary epithelial cells by stimulating autophagy. **a** HPAEpiC were treated with control, LPS, LPS plus HFL-1-exosomes, LPS plus RAPA, LPS plus hucMSCs-exosomes or LPS plus hucMSCs-exosomes and 3-MA, the expression of LC3B was detected by IF. The respective images were shown. **b** The expression of LC3II/I, p62 and Beclin-1 was determined by Western blotting. **c** The autophagy of treated cells was viewed by TEM. **d** The expression of IL-1β, IL-6, IL-17 and MCP-1 in cell culture media was tested by ELISA. *N* = 4.

To test the role of autophagy on ALI, we cultured HPAEpiC and treated with LPS, after 4 h, hucMSCs-exosomes or HFL-1-exosomes was added to the culture medium. The expression of LC3II/I was induced by hucMSCs-exosomes, the similar results were found in the activator of autophagy, RAPA treated cells, while inhibition of autophagy by 3-MA (3-Methyladenine) diminished hucMSCs-exosomes induced autophagy (Fig. 3a, b). The autophagosome also showed the similar results (Fig. 3c). More importantly, hucMSCs-exosomes significantly decreased LPS-induced the expression of inflammatory factors (Fig. 3d). Additionally, activation of autophagy by RAPA suppressed the inflammatory factors as hucMSCs-exosomes did, while 3-MA did not have these effects (Fig. 3d). Meanwhile, chloroquine partially reversed the effect of hucMSCs-exosomes on autophagy (Supplementary Fig. 5a–d). Our results indicated that exosomes released by hucMSCs ameliorated LPS induced inflammation in HPAEpiC by activating autophagy.

### Different expression profile between hucMSCs-exosomes and HFL-1-exosomes

**Fig. 4:**
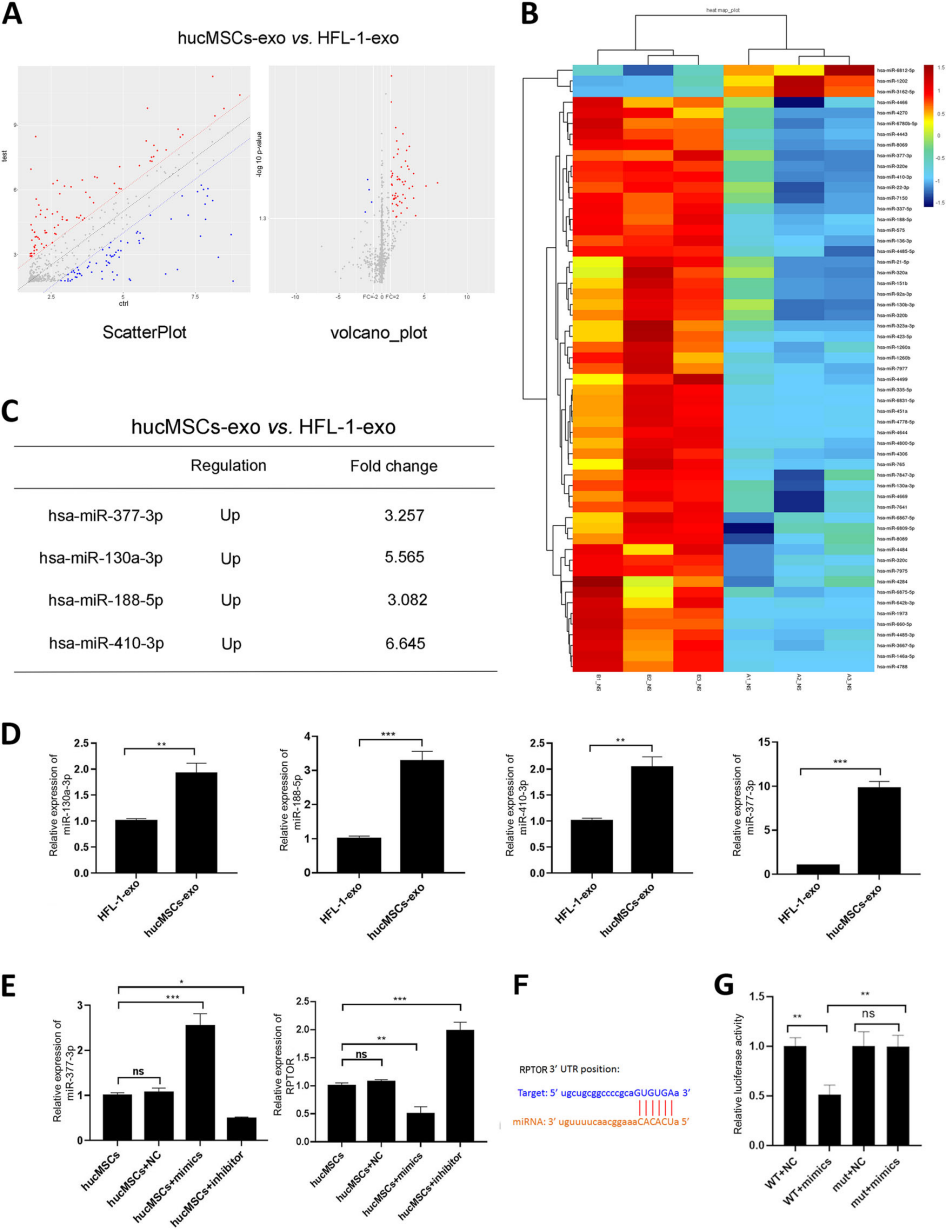
Different expression profile between hucMSCs-exosomes and HFL-1-exosomes. **a** Total RNA from HFL-1-exosomes or hucMSCs-exosomes was extracted by Qiagen Rneasy Mini Kit and subjected to miRNA microarray analysis. **b** The differentially expressed miRNAs were shown by Heat-map. **c** Four up-regulated miRNAs were shown. **d** The expression of miR130a-3p, miR-377-3p, miR-188-5p and miR-410-3p was measured by real-time PCR. **e** hucMSCs were transfected with NC, miR-377-3p mimics or miR-377-3p inhibitor, after 48h, the expression of miR-377-3p was detected by real-time PCR. **f** RPTOR 3′ UTR position was shown. **g** hucMSCs were co-transfected with WT 3′ UTR of RPTOR and NC, WT 3′ UTR of RPTOR and miR-377-3p mimics, mutant form of 3′ UTR of RPTOR and NC or mutant form of 3′ UTR of RPTOR and miR-377-3p mimics, the dual luciferase activity was tested.

To explore the molecular mechanism of hucMSCs-exosomes in protecting ALI in vivo or in vitro, we isolated exosomes from hucMSCs and HFL-1 cells and performed the miRNA microarray to show the differentially expressed miRNA in exosomes (Fig. 4a, b).Among them, the expression of hsa-miR-377-3p, hsa-miR-130a-3p, hsa-miR-188-5p and hsa-miR-410-3p were increased more than 3 folds in hucMSCs-exosomes compared with HFL-1-exosomes (Fig. 4c). We confirmed the expression of these miRNAs by real-time PCR and showed the same results as microassay (Fig. 4d). Moreover, we measured the expression of targets genes of these miRNAs and found that the expression of RHEB, the potential target gene of miR-410-3p and miR-188-5p, were significantly suppressed in hucMSCs-exosomes compared with HFL-1-exosomes, the similar results were found in the expression of AKT2, a potential target gene of miR-130a-3p and the expression of RPTOR, a potential target gene of miR-377-3p (Supplementary Fig. 6a–c).

To further confirm RPTOR was the target gene of miR-377-3p, we activated miR-377-3p by transfected mimics to cells and showed that miR-377-3p mimics significantly decreased RPTOR expression, while inhibition of miR-377-3p by inhibitor enhanced the expression of RPTOR (Fig. 4e). The direct binding of miR-377-3p and 3′ UTR of RPTOR (Fig. 4f) was confirmed by luciferase activity, (Fig. 4g), the luciferase activity was inhibited in wild type 3′ UTR transfected cells, suggesting that RPTOR was a direct target of miR-377-3p.

### MiR-377-3p activates autophagy of HPAEpiC by targeting RPTOR

**Fig. 5:**
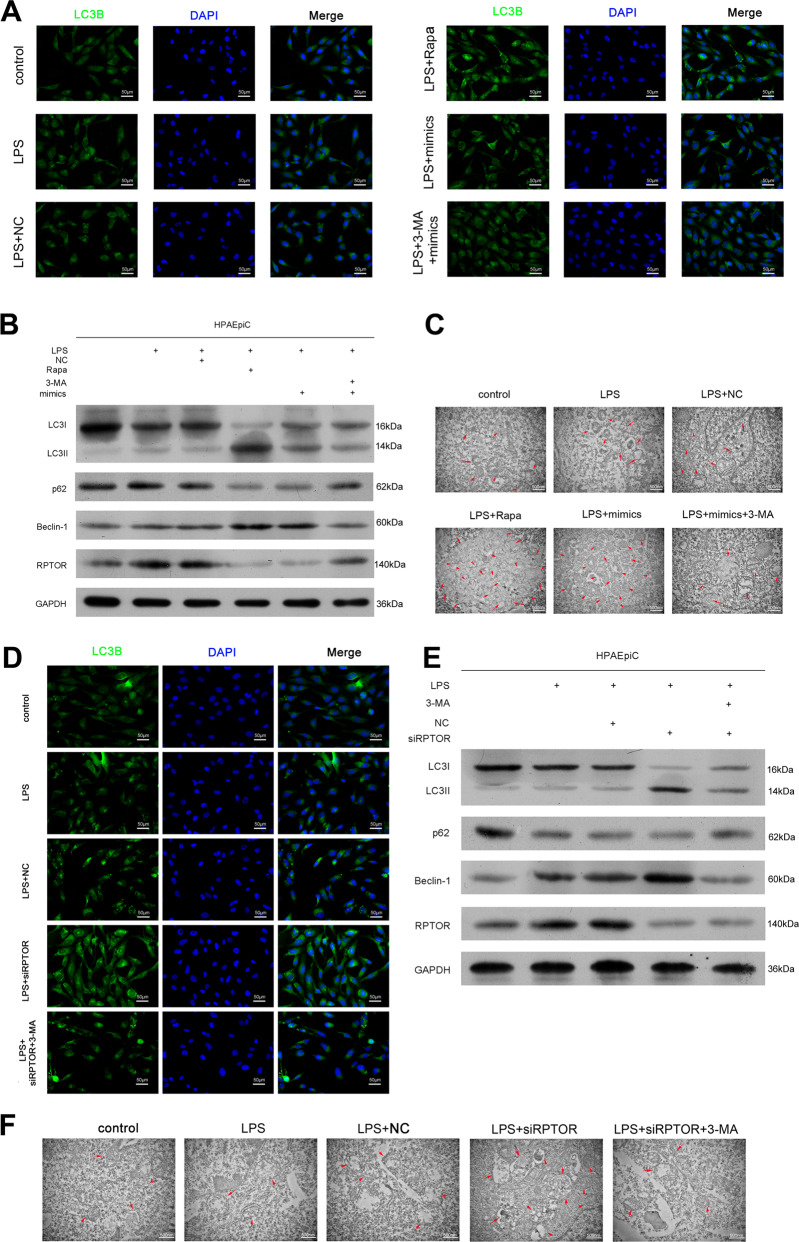
MiR-377-3p activates autophagy of HPAEpiC by targeting RPTOR. **a** HPAEpiC were treated with control, LPS, LPS plus NC, LPS plus RAPA, LPS plus miR-377-3p mimics or LPS plus miR-377-3p mimics and 3-MA, the expression of LC3B was detected by IF. The respective images were shown. **b** The expression of LC3II/I, p62 and Beclin-1 was determined by Western blotting. **c** The autophagy of treated cells was viewed by TEM. **d** HPAEpiC were treated with control, LPS, LPS plus NC, LPS plus RPTOR siRNA or LPS plus RPTOR siRNA and 3-MA, the expression of LC3B was detected by IF. The respective images were shown. **e** The expression of LC3II/I, p62 and Beclin-1 was determined by Western blotting. **f** The autophagy of treated cells was viewed by TEM. *N* = 4.

To determine the active roles of miR-377-3p and its target RPTOR in autophagy, HPAEpiC were transfected with NC or miR-377-3p mimics and treated with LPS, IF, Western blotting and TEM assays showed that miR-377-3p activated autophagy under LPS treatment, as RAPA did (Fig. 5a–c). However, the inhibitor of autophagy, 3-MA alleviated the autophagy caused by miR-377-3p under LPS treatment (Fig. 5a–c). The expression of RPTOR was detected by real-time PCR (Supplementary Fig. 7A). Furthermore, RPTOR was inhibited by siRNA and we found that suppression the target gene of miR-377-3p significantly stimulated autophagy under LPS treatment in HPAEpiC (Fig. 5d–f and Supplementary Fig. 7B). 3-MA also reduced these effects. On the other hand, miR-377-3p was significantly activated by knockdown of RPTOR, and RPTOR was inactivated in the presence of miR-377-3p mimics (Supplementary Fig. 8A). Additionally, the effect of miR-377-3p mimics on autophagy-related proteins was further enhanced by RPTOR silencing (Supplementary Fig. 8B–D). Our data showed that miR-377-3p stimulated autophagy of HPAEpiC under LPS treatment through targeting RPTOR.

### Exosomes released by hucMSCs prevents LPS induced ALI by stimulating autophagy in vivo

**Fig. 6:**
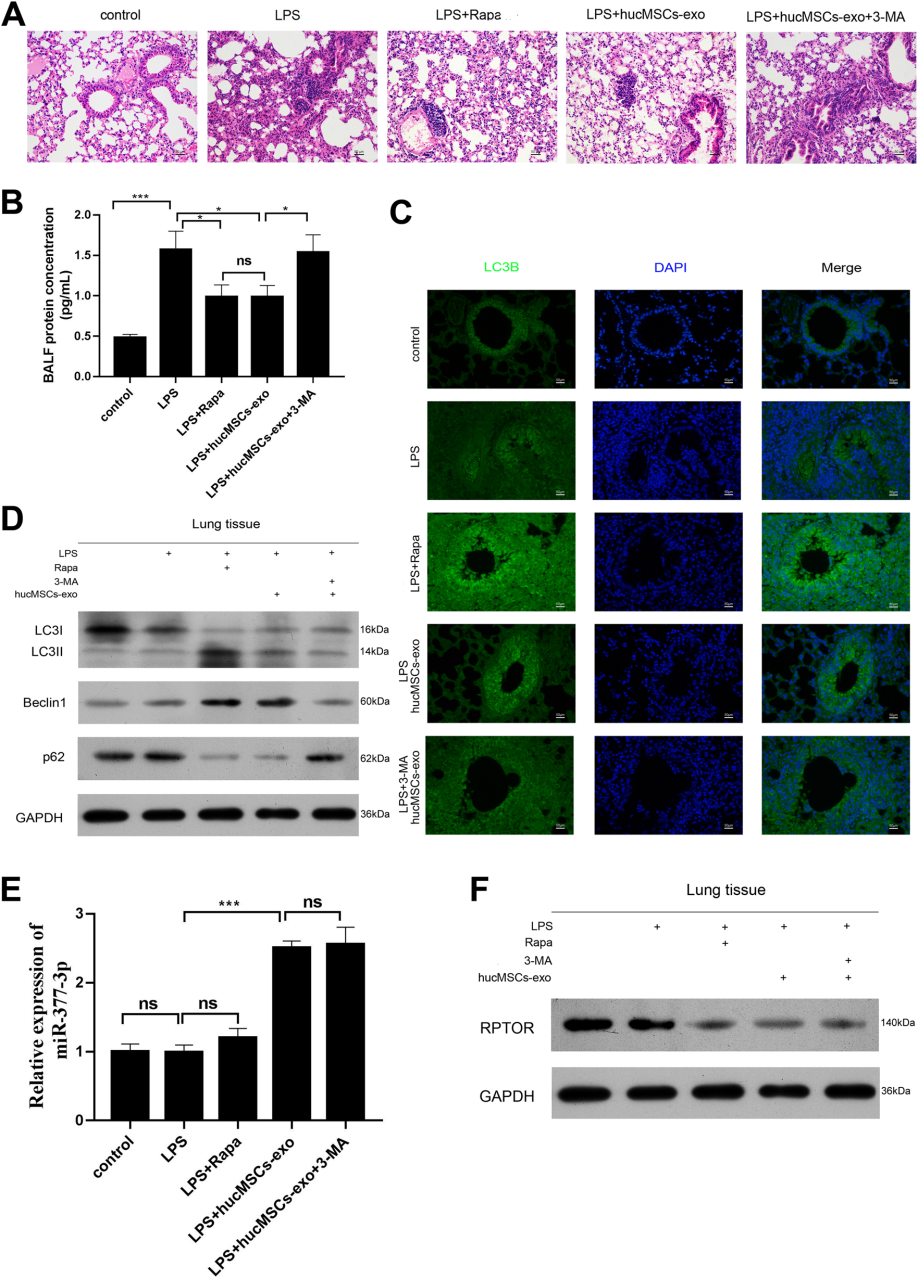
Exosomes secreted by hucMSCs prevents LPS induced ALI by stimulating autophagy in vivo. C57BL/6 mice were intranasal instillated with LPS (1mg/kg weight). **a** After 4h, RAPA, total of 50μg hucMSCs-exosomes or HFL-1-exosomes were subjected to mice through intratracheal instillation, 72h later, the lung tissues were collected and fixed for H&E staining. **b** BALF protein concentration was determined by BCA. *N* = 4. **c** The expression of LC3B was measured by IF. The respective images were shown. **d** The expression of LC3II/I, p62 and Beclin-1 were determined by Western blotting from lung tissues. **e** The expression of miR-377-3p was detected by real-time PCR. **f** The expression of RPTOR was measured by Western blotting.

In LPS induced mice ALI model, the lung morphology and BALF concentration was significantly enhanced, hucMSCs-exosomes alleviated these effects (Fig. 6a, b), however, the inhibitor of autophagy, 3-MA abated hucMSCs-exosomes protective role in ALI (Fig. 6a, b). In order to identify the autophagy in the prevention of LPS induced ALI, LC3II/I expression and other autophagy markers, such as p62 and Beclin-1 was determined by IF or Western blotting, the results showed that hucMSCs-exosomes promoted autophagy, the similar results also shown in RAPA treated mice (Fig. 6c, d). More importantly, 3-MA reduced the autophagy induced by hucMSCs-exosomes (Fig. 6c, d). In addition, we tested the expression of miR-377-3p and its target RPTOR in lung tissues, and found that the expression of miR-377-3p enhanced in hucMSCs-exosomes and LPS co-treated lung tissue compared with LPS alone treated lung tissues, while the expression of RPTOR in hucMSCs-exosomes administrated lung tissues was decreased compared with LPS treated mice (Fig. 6e, f). Besides, hucMSCs-exosomes significantly alleviated the lung tissue injury in LPS-induced mice, while the inhibitory effect of hucMSCs-exosomes was reversed by miR-377-3p inhibitor (Supplementary Fig. 9A). Meanwhile, the pro-inflammatory effect of LPS on mice was inhibited in the presence of hucMSCs-exosomes, which was partially reversed by miR-377-3p inhibitor (Supplementary Fig. 9B). Consistently, the effect of hucMSCs-exosomes on LC3B was partially rescued in the presence of miR-377-3p inhibitor (Supplementary Fig. 9C). These data indicated that activation of autophagy by hucMSCs-exosomes protected LPS induced ALI in vivo.

## Discussion

ALI/ARDS is a severe lung injury with high mortality and is most commonly caused by aspiration, infection, trauma and chemicals^27,28^. Recently, various studies have indicated that administration of hucMSCs show protective roles in ALI^10–12^. In the present study, we found that exosomes released by hucMSCs successfully improved lung morphometry and miR-377-3p in hucMSCs-exosomes played a pivotal role in regulating autophagy by targeting RPTOR, leading to protect LPS induced ALI in vitro and in vivo.

MSCs have shown therapeutic effects in several types of lung disease, including ALI^29^. LPS has been widely used for ALI animal models^30^. A number of studies have proved that MSCs protect against LPS-induced models of ALI^29,31–33^. In addition, MSCs from human umbilical cord are effective in attenuating alveolar fluid clearance and protein permeability in H5N1-associated ALI^34^ and noticeably increase the survival rate of rats suffering from LPS-induced lung injury and significantly reduce systemic and pulmonary inflammation^11^. Here, we showed that after the administration of hucMSCs, the pathological changes, the BALF protein concentration, pulmonary inflammation induced by LPS were relieved. Our results were consistent with previous studies on the beneficial effects of MSCs in resolving ALI, proving that the intra-tracheal injection of hucMSCs had protective effects in attenuating lung injury and inhibiting the lung inflammation of LPS-induced ALI mice.

Recent studies report that autophagy plays an important role in MSC-promoted tissue regeneration^35,36^. Up-regulation of autophagy shows protective roles during ALI^4,6,8^. An autophagy activator, RAPA, significantly inhibited lung inflammation and damage caused by oxidative stress and ALI in mice via activating autophagy^4^ and the inhibition of autophagy significantly exacerbates LPS-induced lung damage^8^. Here, we found that intratracheal instillation of hucMSCs protected LPS induced lung injury by stimulating autophagy. In addition, we showed that exosomes secreted by hucMSCs enhanced autophagy to rescue the lung injury. In HPAEpiC, we confirmed that activation of autophagy by RAPA suppressed the inflammatory response as hucMSCs-exosomes did, indicating that exosomes released by hucMSCs ameliorated LPS induced inflammation in HPAEpiC by activating autophagy. More importantly, our data revealed that 3-MA abated hucMSCs-exosomes protective role in ALI and hucMSCs-exosomes promoted autophagy in LPS induced ALI mice model, the similar results also shown in RAPA treated mice. Our data was consistent with previous studies showing that up-regulation of autophagy protected LPS induced ALI. More importantly, we firstly proved that exosomes secreted by hucMSCs was contributed to the protective effects by stimulating autophagy in ALI. However, there were conflicting results on the role of autophagy in ALI. A part of studies indicate that mice with autophagy deficient macrophages and knockdown of Beclin-1 or Atg5 in alveolar macrophages and mast cells exhibit increased inflammation^3^. Moreover, suppression autophagy augmented LPS induced ALI in HBE cells^5^. The different roles of autophagy in ALI may depend on the cell type affected during lung injury.

Exosomes are small vesicles, which play critical roles in cell communication by transferring RNA, proteins and miRNAs^37^. Recently, more and more studies illustrate that exosomes from MSCs display beneficial roles in ALI. MSC-derived exosomes protect against intestinal ischemia-induced ALI via inhibition of TLR4/NF-κB signaling^38^ and MSCs-exosomes confer protective effects against ALI by inducing the expression of miR-30b-3p^39^. In addition, hucMSCs-exosomes successfully decrease inflammatory factors in rats after burn, and this reduction is reversed by miR-451 expression in hucMSCs-exosomes via the TLR4/NF-κB pathway^40^. We demonstrated in this study that miR-377-3p was significantly increased in hucMSCs-exosomes compared with HFL-1-exosomes through miRNA array assay. Previous studies has demonstrated that miR-377 is down-regulated and functions as a tumor suppressor in several types of cancer cells, such as hepatocellular carcinoma^41^, human clear cellrenal cell carcinoma^42^ and pancreatic cancer^43^ by targeting TIAM1, ETS1 and Pim-3 respectively. In lung cancer, miR-377-5p inhibits cell development and regulated cell cycle distribution and epithelial to mesenchymal transition (EMT) by targeting AKT1^44^ and reduces cell proliferation, promotes apoptosis in non-small lung cancer by targeting CDK6 and AEG-1^45,46^. Additionally, the expression of miR-377 is significantly up-regulated in the plasma of acute graft-versus-host disease patients^46^. In the present study, we proved that the expression of hsa-miR-377-3p was increased more than 3 folds in hucMSCs-exosomes compared with HFL-1-exosomes, and miR-377-3p activated autophagy under LPS treatment. Moreover, suppression the target gene of miR-377-3p, RPTOR significantly stimulated autophagy under LPS treatment in HPAEpiC, indicating miR-377-3p stimulated autophagy of HPAEpiC under LPS treatment through targeting RPTOR. Our results were consistent with previous study showing that inhibition of RPTOR prevented hypoxia-induced lung injury by enhancing autophagy and reducing apoptosis in neonatal mice and *RPTOR*^−/−^ mice decrease apoptosis and improve lung morphometry^47^. Moreover, a previous study found that RPTOR was a target of miR-155 and elicits a fibrotic phenotype of cystic fibrosis lung epithelium^48^. In our ALI mice model, the expression of miR-377-3p enhanced in hucMSCs-exosomes and LPS co-treated lung tissues compared with LPS alone treated lung tissues, while the expression of RPTOR in hucMSCs-exosomes administrated lung tissues was decreased compared with LPS treated mice, indicating that activation of autophagy by hucMSCs-exosomes protected LPS induced ALI in vivo. Frankly speaking, this study only focused on one miRNA. Since various miRNAs are involved in ALI^49^, we will investigate the effect of other miRNAs on ALI in the future.

In conclusion, our data demonstrated that exosomes released by hucMSCs played an essential role in protecting LPS induced ALI by inducing autophagy. Furthermore, analysis of miRNA sequencing and luciferase assay showed that miR-377-3p regulating autophagy by targeting RPTOR. Therefore, enhancing the function of autophagy could provide a promising therapeutic approach, enabling the treatment of an overwhelming autophagy response.

## Supplementary information

Sequencing data

Supplementary Figure legends

Supplementary Figure 1

Supplementary Figure 2

Supplementary Figure 3

Supplementary Figure 4

Supplementary Figure 5

Supplementary Figure 6

Supplementary Figure 7

Supplementary Figure 8

Supplementary Figure 9
