# Correction: MicroRNA-377-3p released by mesenchymal stem cell exosomes ameliorates lipopolysaccharide-induced acute lung injury by targeting RPTOR to induce autophagy

**DOI:** 10.1038/s41419-020-02976-y

**Published:** 2020-09-12

**Authors:** Xuxia Wei, Xiaomeng Yi, Haijin Lv, Xin Sui, Pinglan Lu, Lijuan Li, Yuling An, Yang Yang, Huimin Yi, Guihua Chen

**Affiliations:** 1 Surgical and Transplant Intensive Care Unit, The Third Affiliated Hospital of Sun Yat-Sen University, No. 600, Tianhe Road, Tianhe District, Guangzhou, 510630 Guangdong People’s Republic of China; 2 Department of Hepatic Surgery and Liver transplantation Center of the Third Affiliated Hospital, Organ Transplantation Institute, Sun Yat-sen University; Organ Transplantation Research Center of Guangdong Province, Guangdong province engineering laboratory for transplantation medicine, The Third Affiliated Hospital of Sun Yat-Sen University, No. 600, Tianhe Road, Tianhe District, Guangzhou, 510630 Guangdong People’s Republic of China

**Keywords:** Mesenchymal stem cells, Respiratory tract diseases

Correction to: *Cell Death and Disease*

10.1038/s41419-020-02857-4 published online 19 August 2020

The original version of this Article omitted the following from the Acknowledgements:

This work was supported by National 13th Five-Year Science and Technology Plan Major Projects of China (2017ZX10203205-006-001); National Key R&D Plan (2017YFA0104304); National Natural Science Foundation of China (81770648 81972286); Guangdong Natural Science Foundation (2015A030312013, 2018A0303130305); Science and Technology Program of Guangdong Province (2017B020209004, 20169013, 2017B030314027). This has now been corrected in both the PDF and HTML versions of the Article.

