# Supplementary material for: MicroRNA-377-3p released by mesenchymal stem cell exosomes ameliorates lipopolysaccharide-induced acute lung injury by targeting RPTOR to induce autophagy
Source: Cell Death Dis. 2020 Aug 19;11(8):657. doi: 10.1038/s41419-020-02857-4 (PMC7438519; doi:10.1038/s41419-020-02857-4)
Supplement: Supplementary file 2 — Supplementary Figure legends [file 41419_2020_2857_MOESM2_ESM.pdf]

Supplementary figure 1 Identification of hucMSCs

A. hucMSCs were subjected to flow cytometry by incubating with CD73, CD90, CD105, CD19, CD34 or HLA-DR antibody. The cell performance was shown. B. hucMSCs of the third passages were cultured in osteogenic differentiation or adipogenic differentiation medium for 3 weeks. Cells were fixed stained with oil red O or alizarin red S. The phenotype was shown.

Supplementary figure 2 Autophagy takes part in the protection of hucMSCs in LPS induced ALI

A. hucMSCs were transfected with negative control (NC) shRNA, Rab27a-1 or Rab27a-2 shRNA, after 48 hours, cells were collected for Western blotting. B. C57BL/6 mice were intranasal instillated with LPS (1 mg/kg weight). After 4 hours, total of 2×10^5 hucMSCs-NC shRNA or hucMSCs-Rab27a-2 shRNA were subjected to mice through intratracheal instillation, 72 hours later, the lung tissues were collected and fixed for H&E staining. N=4. C. BALF protein concentration was determined by BCA. N=4. D. The expression of IL-1β, IL-6, IL-17 and MCP-1 in BALF was tested by ELISA. N=4. E. The expression of LC3B was measured by IF. The respective images were shown. F. The expression of LC3II/I, p62 and Beclin-1 were determined by Western blotting from lung tissues.

Supplementary Figure3 The lung histology score of HE stain

Supplementary Figure 4 The particle sizes of exosomes

A. The concentration of particles in exosomes was calculated. B. Three-dimensional

map of particles was presented.

Supplementary Figure 5 Chloroquine partially reversed the effect of hucMSCs-exosomes on autophagy

A The protein expressions of LC3I, LC3II, p62 and Beclin-1 in HPAEpiC were detected by western blot. B The expression of IL-1β, IL-6, IL-17 and MCP-1 in cell culture media was tested by ELISA. C The expression of LC3B was detected by immunofluorescence staining. D TEM was used to observe the exosomes. N=4.

Supplementary Figure 6 mRNA expression of miRNAs targets

A-C. RNA from HFL-1 cells and hucMSCs were extracted and the expression of RPTOR, AKT1 and Rheb was detected by real-time PCR.

Supplementary Figure 7 mRNA expression of RPTOR

A-B. Cells were treated as indicated, the expression of RPTOR was tested by real-time PCR.

Supplementary Figure 8 Silencing of RPTOR further enhanced the effect of miR-377-3p on autophagy-related proteins

A. The expression of miR-377-3p and RPTOR was detected by qRT-PCR. B. The protein expressions of RPTOR, LC3, P62 and Beclin-1 were measured by western blot. C. The expression of L3B was detected by immunofluorescence staining. D. TEM was used to observe the cell autophagy.

Supplementary Figure 9 The effect of hucMSCs-exosomes on ALI mice was partially reversed by miR-377-3p inhibitor

A HE staining was performed to observe the lung injury. B The levels of IL-6, IL-1β, MCP-1 and IL-17 were detected by ELISA. C The expression of LC3B was detected by IF.
