# Supplementary figures and images for: MicroRNA-377-3p released by mesenchymal stem cell exosomes ameliorates lipopolysaccharide-induced acute lung injury by targeting RPTOR to induce autophagy
Source: Cell Death Dis. 2020 Aug 19;11(8):657. doi: 10.1038/s41419-020-02857-4 (PMC7438519; doi:10.1038/s41419-020-02857-4)

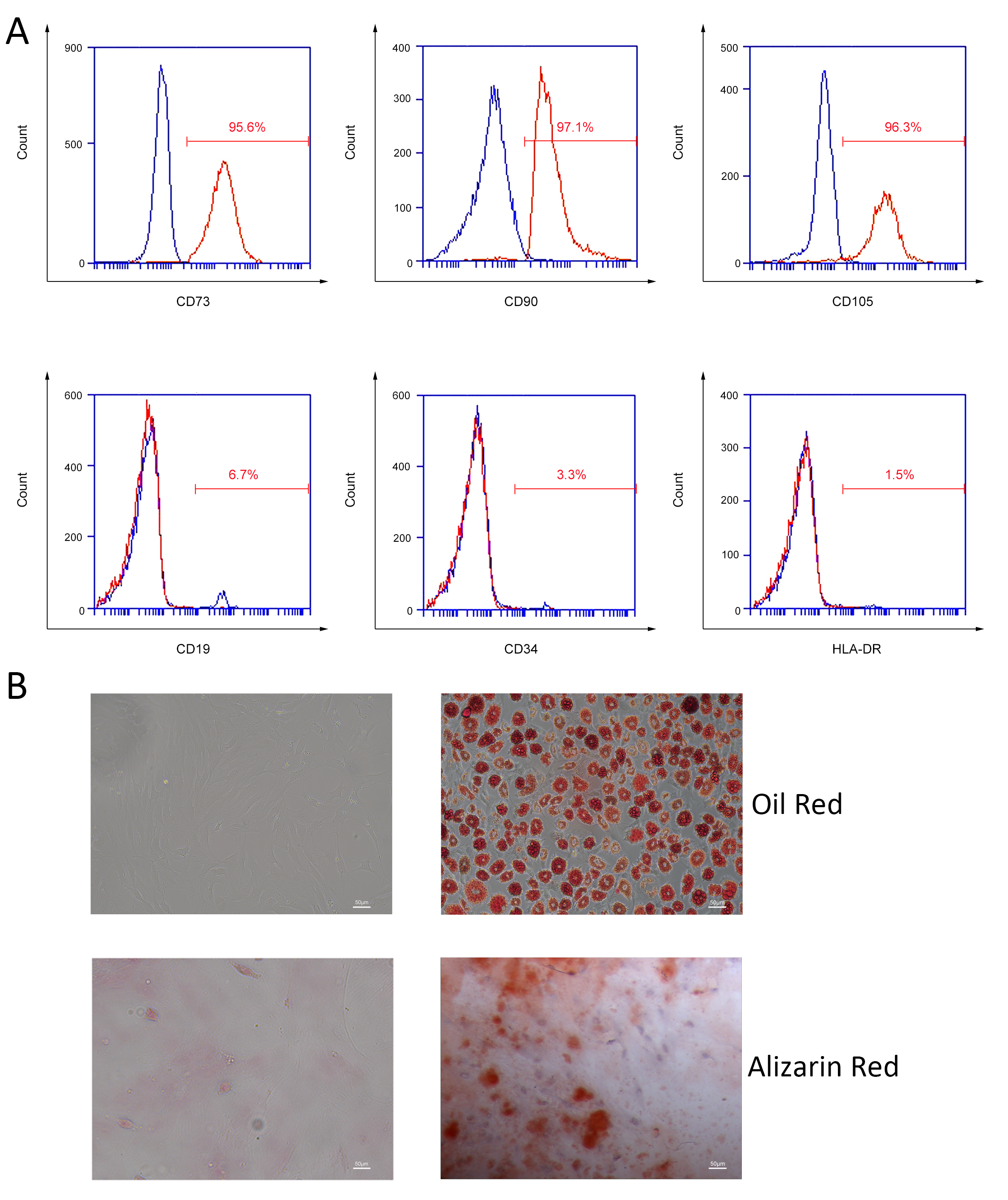

Supplement: Supplementary file 3 — Supplementary Figure 1 [file 41419_2020_2857_MOESM3_ESM.png]

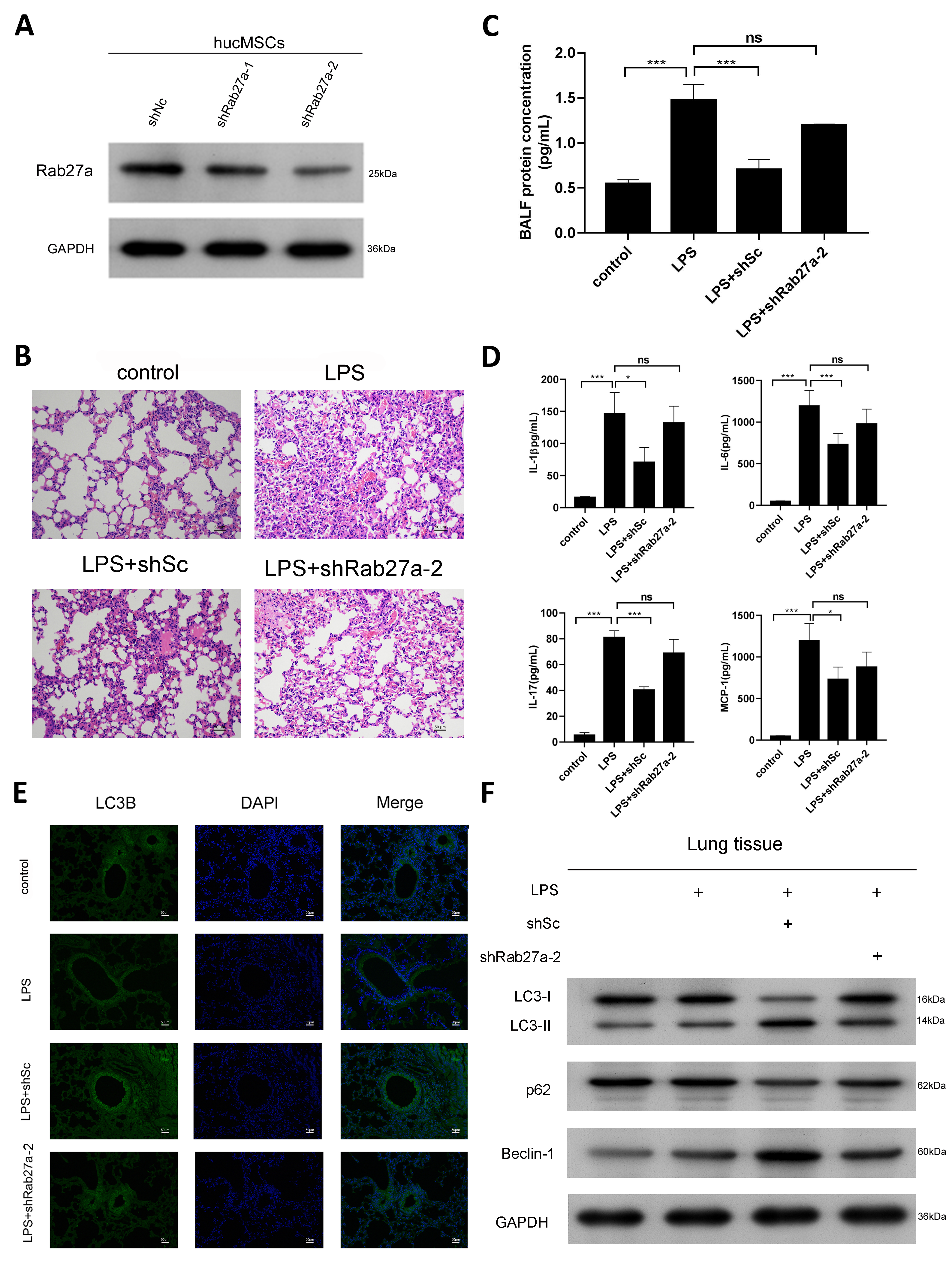

Supplement: Supplementary file 4 — Supplementary Figure 2 [file 41419_2020_2857_MOESM4_ESM.png]

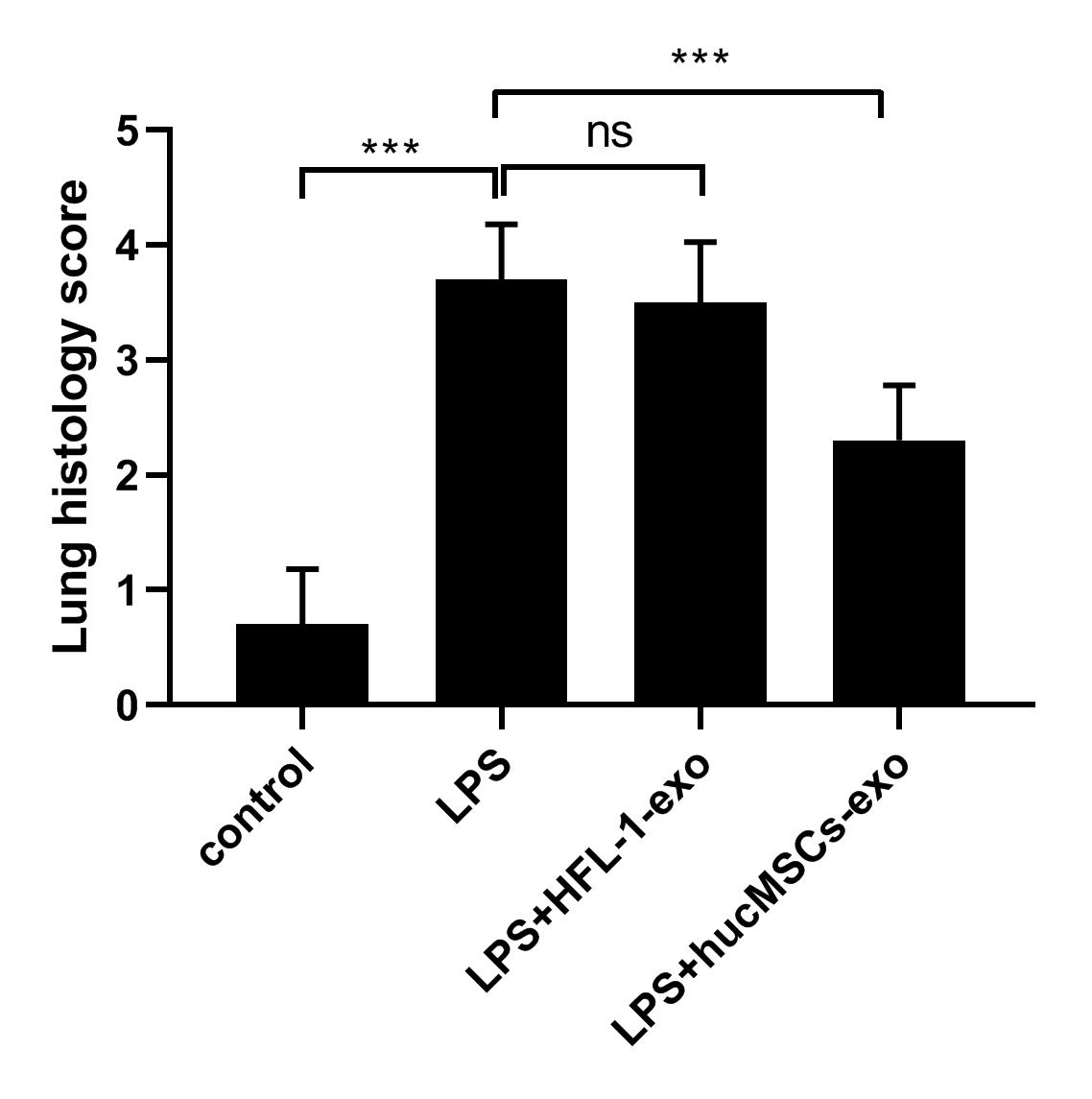

Supplement: Supplementary file 5 — Supplementary Figure 3 [file 41419_2020_2857_MOESM5_ESM.png]

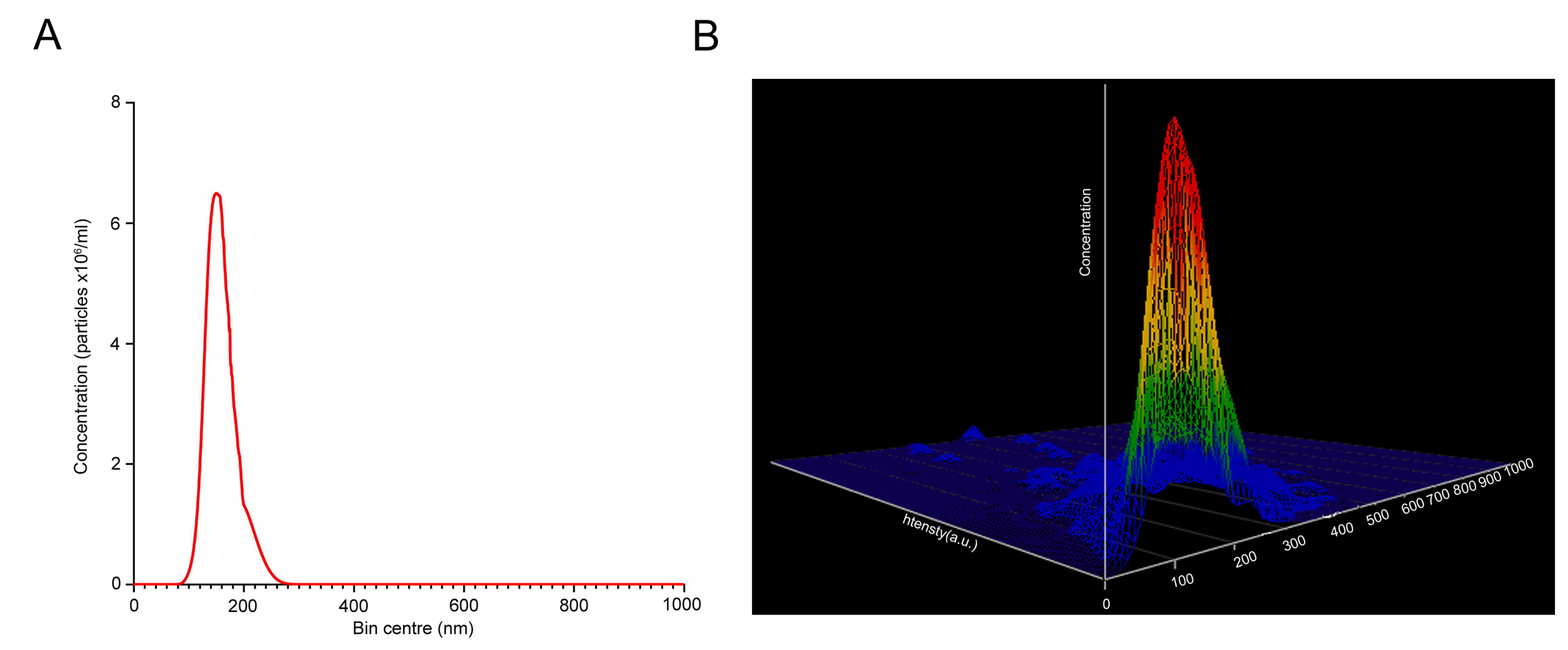

Supplement: Supplementary file 6 — Supplementary Figure 4 [file 41419_2020_2857_MOESM6_ESM.png]

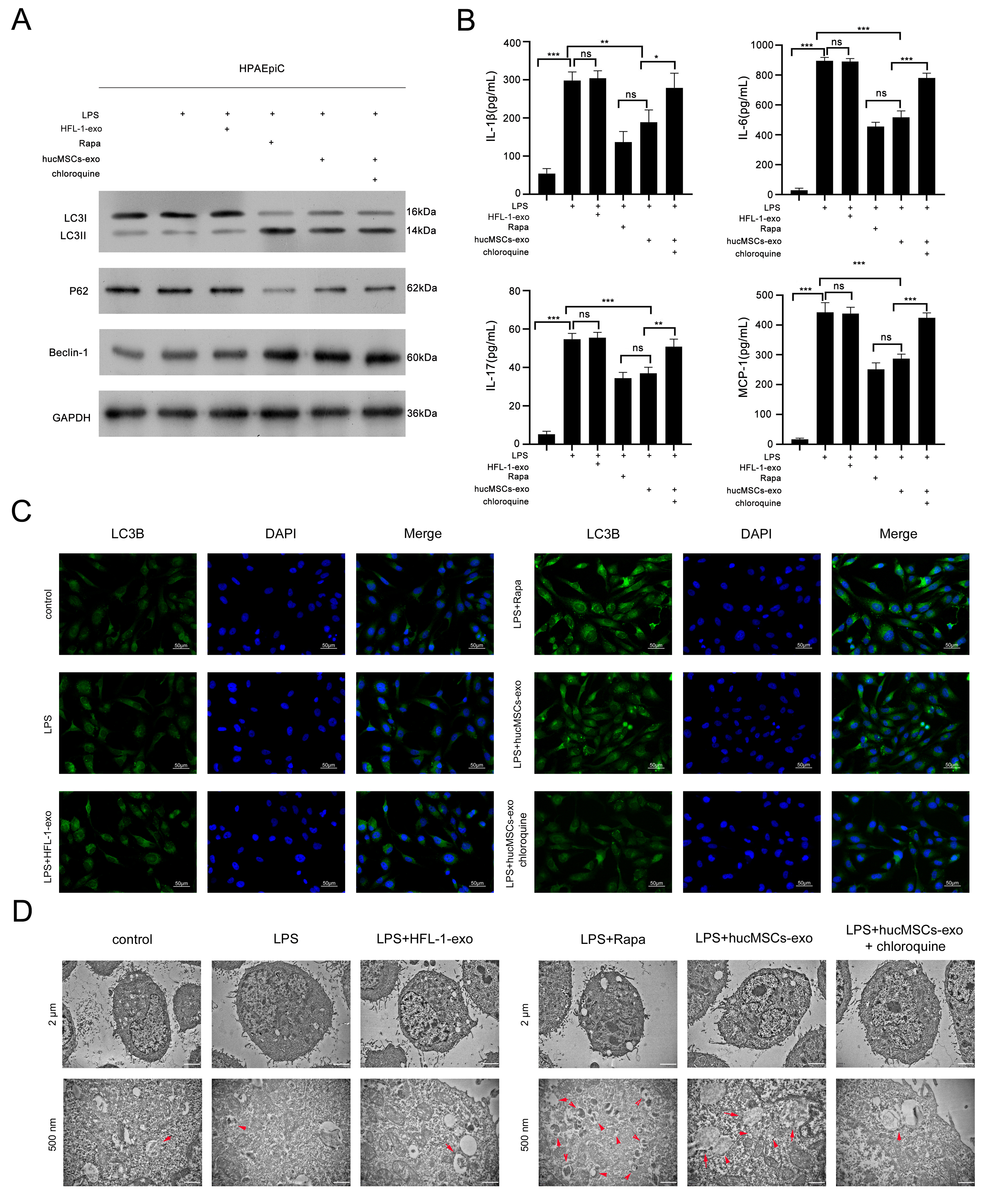

Supplement: Supplementary file 7 — Supplementary Figure 5 [file 41419_2020_2857_MOESM7_ESM.png]

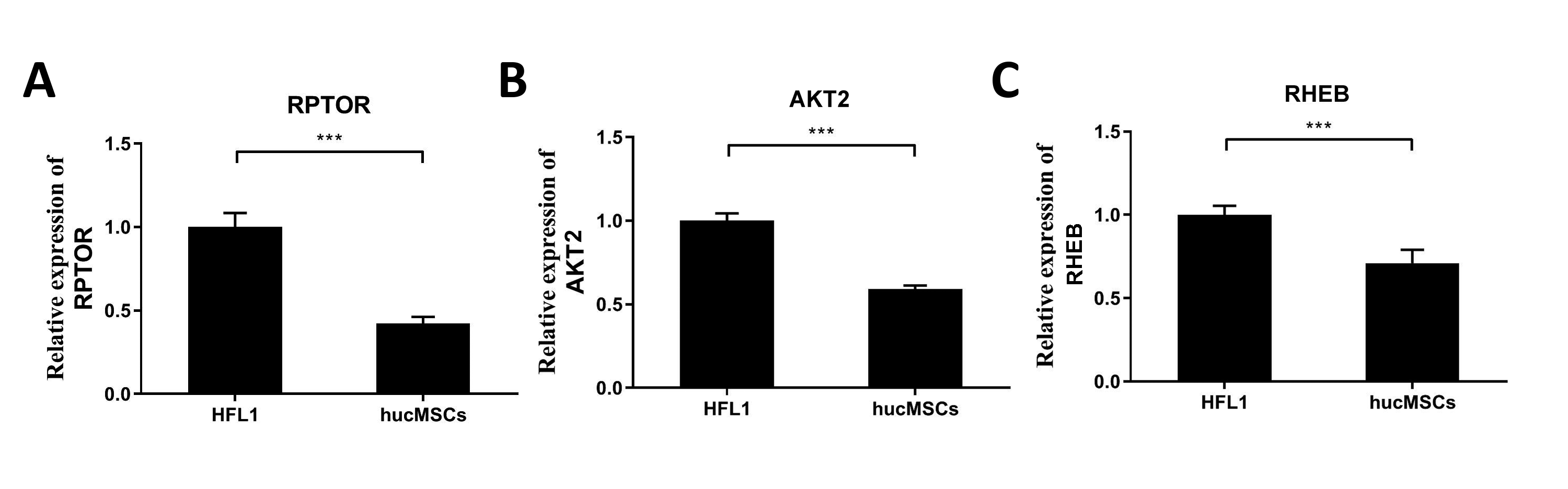

Supplement: Supplementary file 8 — Supplementary Figure 6 [file 41419_2020_2857_MOESM8_ESM.png]

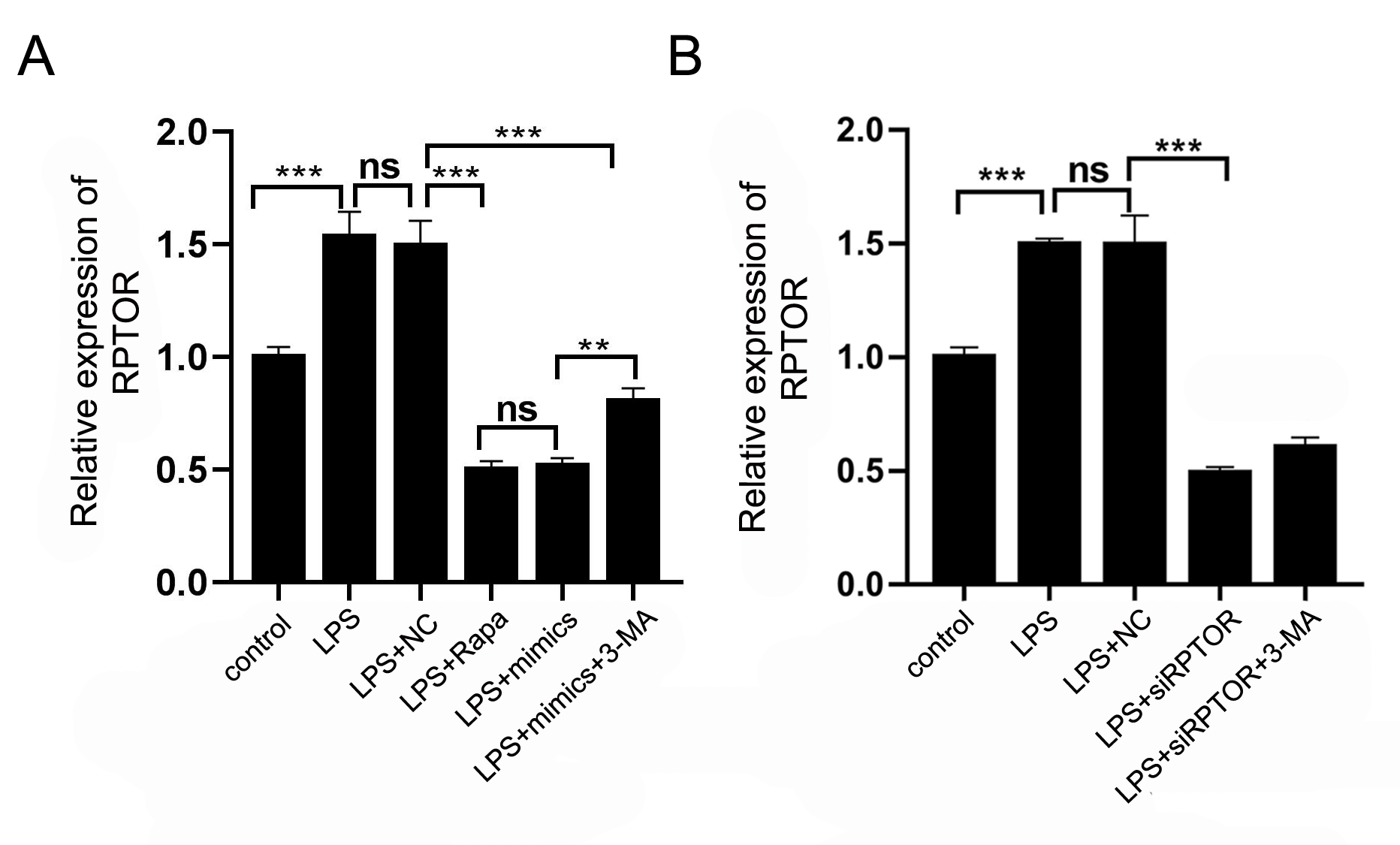

Supplement: Supplementary file 9 — Supplementary Figure 7 [file 41419_2020_2857_MOESM9_ESM.png]

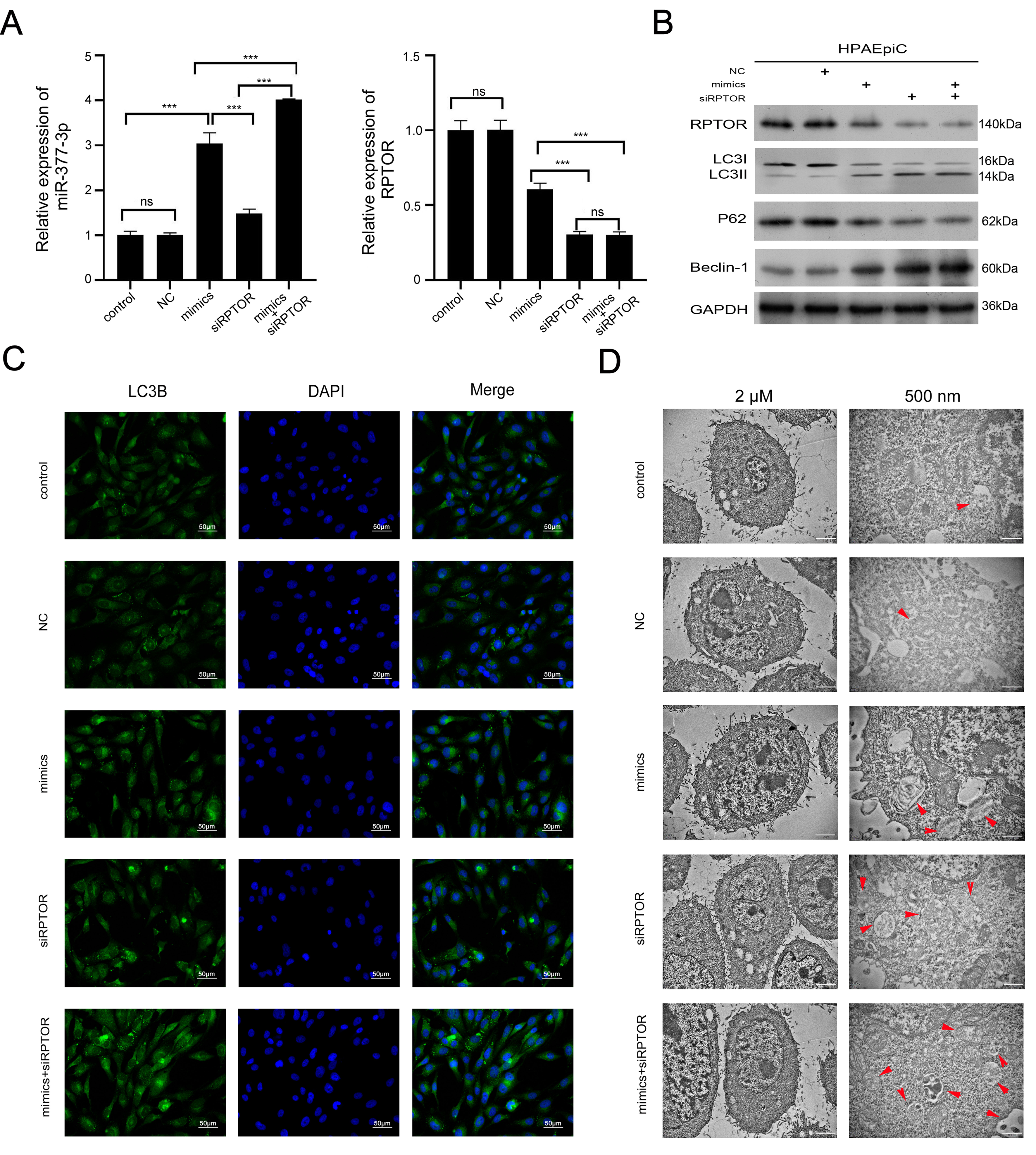

Supplement: Supplementary file 10 — Supplementary Figure 8 [file 41419_2020_2857_MOESM10_ESM.png]

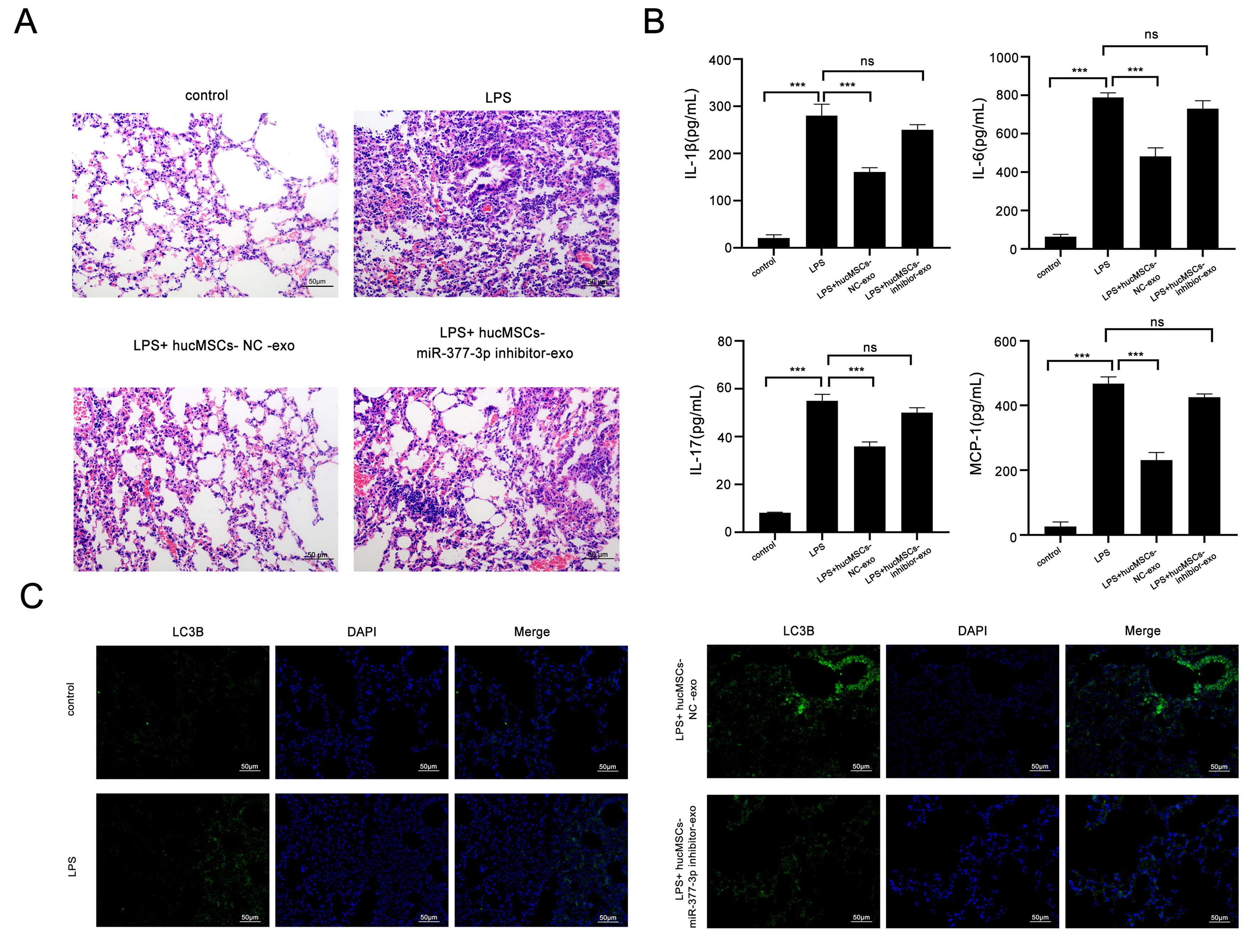

Supplement: Supplementary file 11 — Supplementary Figure 9 [file 41419_2020_2857_MOESM11_ESM.png]
